# Sports teams as complex adaptive systems: manipulating player numbers shapes behaviours during football small-sided games

**DOI:** 10.1186/s40064-016-1813-5

**Published:** 2016-02-27

**Authors:** Pedro Silva, Luís Vilar, Keith Davids, Duarte Araújo, Júlio Garganta

**Affiliations:** FC Zenit, St. Petersburg, Russia; Centre for Research, Education, Innovation and Intervention in Sport (CIFI2D), Faculty of Sport, Universidade do Porto, Rua Dr. Plácido Costa, 91, 4200-450 Porto, Portugal; Escola de Turismo, Desporto e Hospitalidade, Universidade Europeia, Lisboa, Portugal; CIPER, Faculdade de Motricidade Humana, Universidade de Lisboa, Cruz Quebrada Dafundo, Portugal; Centre for Sports Engineering Research, Sheffield Hallam University, Sheffield, UK; FidiPro Programme, University of Jyväskylä, Jyväskylä, Finland

**Keywords:** Small-sided and conditioned games, Team games as complex adaptive systems, Relative space per player, Emergent behaviours, Degeneracy

## Abstract

Small-sided and conditioned games (SSCGs) in sport have been modelled as complex adaptive systems. Research has shown that the relative space per player (RSP) formulated in SSCGs can impact on emergent tactical behaviours. In this study we adopted a systems orientation to analyse how different RSP values, obtained through manipulations of player numbers, influenced four measures of interpersonal coordination observed during performance in SSCGs. For this purpose we calculated positional data (GPS 15 Hz) from ten U-15 football players performing in three SSCGs varying in player numbers (3v3, 4v4 and 5v5). Key measures of SSCG system behaviours included values of (1) players’ dispersion, (2) teams’ separateness, (3) coupling strength and time delays between participants’ emerging movements, respectively. Results showed that values of participants’ dispersion increased, but the teams’ separateness remained identical across treatments. Coupling strength and time delay also showed consistent values across SSCGs. These results exemplified how complex adaptive systems, like football teams, can harness inherent degeneracy to maintain similar team spatial–temporal relations with opponents through changes in inter-individual coordination modes (i.e., players’ dispersion). The results imply that different team behaviours might emerge at different ratios of field dimension/player numbers. Therefore, sport pedagogists should carefully evaluate the effects of changing RSP in SSCGs as a way of promoting increased or decreased pressure on players.

## Background

Team ball sports like association football are considered complex adaptive systems affording the emergence of rich patterns of behaviour from players in dynamically changing environments (Passos et al. 2008b; Duarte et al. 2013). Understanding coordination dynamics in social complex systems like team games is not just predicated on studying the individual motions of component parts (i.e. the competing and cooperating players), as highlighted by performance analysts. Rather research has shown how coordination processes in team sports lead to the emergence of functional, spatiotemporal, patterned relations between performers, as they adapt to the movements of teammates and opponents during performance. Analysis of the dynamics of coordination between performers can characterise the emergent couplings between different parts of the complex adaptive system (Davids et al. 2005; Balague et al. 2013).

Developing understanding in this area of work is not just of theoretical value in sport science but is crucial for designing effective practice simulations in team sports since tactical determinants reflect the behavioural co-adaptations of individual players to specific task constraints (Silva et al. 2014). For instance, Fradua et al. (2013) have analysed the effective playing space of football teams during competitive fixtures in an attempt to specify similar playing areas for practice tasks during small-sided and conditioned games (SSCGs).

In the past decade a large number of studies have adopted a complex systems orientation to focus on the utility of SSCGs in team sports like association football (for a review see Davids et al. 2013). Despite the recognition that SSCGs offer a potential stimulus for the development of decision-making and individual tactical abilities, there have been very few attempts to capture the tactical coordination processes emerging between players and/or groups of players while undertaking such practice tasks (Vilar et al. 2014; Serra-Olivares et al. 2015a, b). Co-adaptation occurs through the continuous spatial and temporal re-organization of small sub-groups of attackers and defenders coordinating their actions together to achieve common performance goals. At a collective level, emergent co-adaptive behaviours between individuals can be harnessed during SSCGs if players are provided with opportunities, through changing task constraints, to continuously adjust their actions relative to emerging behaviours of others (Vilar et al. 2012). Such dynamic interpersonal interactions include players continuously (re) orienting themselves in relation to constantly changing positions, directions, angles of teammates and opponents, as well as locations of ball, field/court markings and target areas (goal, basket, try-line), during practice (Duarte et al. 2012). These intertwined relations invite (collective) actions (Withagen et al. 2012) that sustain team coordination under the constraints of dynamic performance environments like team sports (Silva et al. 2013).

One of the most important characteristics of biological complex systems, like football teams, is the ability to degenerate with their multitudinous degrees of freedom to satisfy different constraints on behaviour (Davids and Araújo 2010; Edelman and Gally 2001). Neurobiological system degeneracy is referred as the ability of elements that are structurally different to perform the same function or yield the same output (Davids and Glazier 2010; Seifert et al. 2013). This means that similar team behaviours can result from distinct interpersonal coordination modes through the exploitation of system degeneracy to explore the same functional solutions. In this sense, manipulations to key task constraints during practice, targeting the exquisitively timed, relative spatial (re) positioning of attackers and defenders can be continuously used to shape skilled performance and tactical behaviours through co-adaptation (Chow et al. 2006; Davids et al. 2013).

Many recent studies have observed the dynamics of interpersonal interactions in team sports like football and futsal, shaped by key informational constraints such as values of interpersonal distances between performers (Duarte et al. 2010), ball location on-field (Vilar et al. 2012; Travassos et al. 2012) and goal location (Headrick et al. 2012; Travassos et al. 2014). There is a need to understand whether modifications to the task constraints of SSCGs can lead to emergence of specific tactical behaviours of players and teams, by altering the presence and availability of such informational constraints during training.

From the perspective of the complexity sciences, the location of the system on the performance spectrum at a given moment is influenced by the constraints that are acting upon it (Davids et al. 2003, 2008). It has been shown that modifications in pitch dimensions and the number of players involved during SSCGs can affect a practice task’s technical and physical demands (Dellal et al. 2012) and, most importantly, the tactical aspects of performance (Platt et al. 2001). In this sense, the relative space per player (RSP, here defined as the total playing area divided by the number of players) is a key manipulable constraint that may be affected by the emergent relations between the number of players and pitch dimensions involved during practice. The tactical implications of different RSPs have received very little attention, however.

Nonetheless, both coaching knowledge (e.g., Lucchesi 2002), and research on team sports (e.g., Frencken et al. 2013) have suggested that different RSPs can shape the spatial–temporal interactions between teammates and opponents. In previous work, Frencken and colleagues (Frencken et al. 2013) observed significantly different inter-team lateral and longitudinal distance values to arise from different RSPs. Shorter pitches (i.e., lower RSPs through exclusive manipulation of pitch dimensions) resulted in smaller values of inter-team distances. In this sense, lower RSPs are expected to restrict space between opposing players and vice versa (Lucchesi 2002) and, thus, promote different playing styles during attacking and defensive sub-phases of play. Following previous insights from research on SSCGs (e.g., Folgado et al. 2012) such styles might be related to different spatial–temporal relations established between opposing teams, influencing: (1) the players’ dispersion on-field, (2) the space separating players from their nearest opponents, (3) the relative co-dependence of teams on each other’s movements, and (4), the time taken for one team to respond to the emerging movements of the other.

In this study we aimed to extend knowledge on how practice in SSCGs shapes emergent tactical behaviours by analysing the influence of different values of RSP on the spatial–temporal characteristics of inter-team coordination. Here, we manipulated RSP through modifications of player numbers, while keeping pitch dimensions constant. Inter-team coordination processes were studied through analysis of players’ dispersions, the free space separating each player from his nearest opponent and the coupling strength of each team’s movements and associated response time delays. Although the extant literature on manipulations of RSP is sparse, following insights from the limited previous work (e.g., Frencken et al. 2013), it was hypothesized that, as values of RSP decrease, the space between nearest opponents and the response delay between teams’ co-adaptive movements would also decrease, while the strength of their co-dependence and the players’ dispersion values would increase.

## Methods

### Participants

Ten players in an under-15 years football squad (*M* ± *SD*, age: 13.6 ± 0.52 years), competing at a regional-level, participated in this experiment (*M* ± *SD*, playing and training experience: 4.10 ± 1.77 years). The players were chosen based on their experience in official competitive fixtures. The players with more completed competitive fixtures in the team were chosen to participate in this study (goalkeepers excluded). Their legal tutors provided signed and informed consent authorizing their participation. All procedures were in accordance with the ethical standards of the Faculty of Sports of Porto University.

### Small-sided games

Participants were assigned to two technically and equivalent teams of five players (two defenders, two midfielders and one striker in each team) by their coach (possessing a UEFA-Basic diploma and eight years of coaching experience in youth football) and performed in three SSCGs in the following order: 3v3, 4v4 and 5v5 played without goalkeepers (in the 3v3 only six players have participated—one defender, one midfielder and one striker for each team—and in the 4v4 only eight players have participated—one defender, two midfielders and a striker for each team). Pitch dimension was maintained at 36 × 28 m (length × width) (as in the study of Frencken et al. (2011) using similar SSCGS) and therefore, the RSP differed across SSCGs format (168 m^2^ on 3v3, ≈126 m^2^ on 4v4 and ≈100.8 m^2^ on 5v5). Two mini-goals (120 × 80 cm, width × height) were placed on each goal line, 7 m away from each pitch corner. The treatment of playing conditions was counterbalanced for individuals and the work/rest ratio was 1:1 (5-min of play and 5-min of recovery). The SSCGs time duration was set by the coach based on the usual duration of other types of SSCGs that was implemented in training sessions. The coach did not provided any type of instructions or encouragement during the SSCGs.

Displacing several balls around the pitch that were promptly provided to the players by the coach or the two experimenters minimized game stoppages.

During the recovery periods, players rehydrated and recovered actively at will through low intensity activities (e.g., performing short passes in pairs). All trials were played according to official laws of Association Football with the exception of the offside law, that was not applied.

### Data collection

Each participant carried a global positioning measure tracking device (SPI Pro, GPSports, Canberra, Australia) that recorded his 2D positional coordinates, at a sampling frequency rate of 15 Hz. The reliability of such type of devices has been well documented elsewhere (Johnston et al. 2013; Coutts and Duffield 2010).

The pitch was calibrated with the coordinates of four GPS devices stationed in each corner of the pitch for about 2 min. The absolute coordinates of each corner were calculated as the median of the recorded time series, providing measurements that were robust to the typical fluctuations of the GPS signals. These absolute positions were used to set the Cartesian coordinate systems for each pitch, with the origin placed at the pitch centre. Longitudinal and latitudinal (spherical) coordinates were converted to Euclidean (planar) coordinates using the Haversine formula (Sinnott 1984). Fluctuations in the players’ positioning were reduced using a moving average filter with a time scale of 0.2 s and data resampling was employed to synchronize the time series of all players within each game.

### Variables

Position data were used to calculate the players’ dispersion, teams’ separateness, and time delay and coupling strength between teams’ movements. The players’ dispersion is a stretch index measure of all players on the field and it was computed by averaging their distances to the total match centroid (i.e., the geometrical centre of both teams’ players). We calculated the overall players’ dispersion and the dispersion in the goal-to-goal and side-to-side directions of the field.

Teams’ separateness is a measure of the degree of free movement each team has available and it was calculated for the 3v3, 4v4 and 5v5 treatments by organizing the distances between opponent players in a pair-wise distance matrix M(t) of order 9 (3 × 3 players), 16 (4 × 4 players) and 25 (5 × 5 players), respectively. Because each treatment uses a different number of players, in this study the teams’ separateness was defined as the average distance between all players and their closest opponent and was interpreted as the average radius of action free of opponents. The players’ dispersion, players’ dispersion in the goal-to-goal and side-to-side directions and teams’ separateness have units of meters (m).

The time delay between teams’ movements was calculated by lagging the teams’ centroids in time, relative to one another, for maximal agreement reported through the highest correlation coefficient values obtained between the centroids coordinates. To this effect, a windowed cross-correlation technique with overlapping time windows that covered the whole time series data was used, producing a moving estimate of association and lag (Boker et al. 2002). The maximum lags (i.e., the lags registering the highest positive *r* values) were considered to represent the time delay, in seconds, between the two centroids. A window size of 20-s was used because it was reported as the minimum amount of time necessary for both teams to have had ball possession, in all treatments. A maximum lag range of 5-s was chosen based on the experiential knowledge of a panel of five qualified youth player coaches (coaching experience: 11.6 ± 3.9 years in youth Football) as a reasonable value for the maximum delay between two teams’ movements during this type of SSCGs. The teams’ coupling strength was obtained by simply extracting the *r* values at the zero-lag. The coupling strength represents the degree of coordination or synchronization between the teams’ movements. Both time delay and coupling strength were calculated for the goal-to-goal and side-to-side directions.

It is important to note that it was not the aim of this study to analyse *the direction* of the lags, i.e., to understand which team was leading which at specific moments of the match. Thus, all max lags differing from zero were considered positive lags, independent of the team that was temporally leading the competitive interactions.

All variables are synthesized in Table 1.

**Table 1:** Synthesis of the variables used

Players’ dispersion (overall)	Stretch index measure of all players on the field. It provides a measure of compactness of the team
Players’ dispersion in the goal-to-goal direction	Stretch index measure of all players on the field in the longitudinal direction
Players’ dispersion in the side-to-side direction	Stretch index measure of all players on the field in the lateral direction
Teams’ separateness	Measure of the degree of free movement each team has available. It provides an estimate of the amount of space separating the players of both teams
Time delay between teams’ movements in the goal-to-goal direction	Quantifies the existing time delay between both teams’ movements using each team centroid as a reference. It represents the delay of teams in adjusting to each other’s movements in the longitudinal direction of the field
Time delay between teams’ movements in the side-to-side direction	Quantifies the existing time delay between both teams’ movements using each team centroid as a reference. It represents the delay of teams in adjusting to each other’s movements in the lateral direction of the field
Coupling strength in the goal-to-goal direction	It measures the degree of coordination or synchronization between the teams’ movements in the longitudinal direction of the field
Coupling strength in the side-to-side direction	It measures the degree of coordination or synchronization between the teams’ movements in the lateral direction of the field

### Data analysis

For each variable we counted the number of time points performed under specific magnitude intervals. The players’ dispersion, players’ dispersion in the goal-to-goal direction, players’ dispersion in the side-to-side direction and teams’ separateness were divided in 1 m intervals from 0 to 16 m. The max lags extracted from the time delay in the goal-to-goal and side-to-side directions were distributed across 10 intervals of 0.5-s from 0 to 5-s and the *r* values of the coupling strength in the goal-to-goal and side-to-side directions were distributed across 10 intervals of 0.1 from 0 to 1 (it were only considered positive *r* values).

All variables were then analysed for consistency across SSCGs using intraclass correlation analysis (ICC). We applied an ICC two-way mixed model with single measures (ICC 3,1) and the following convention has been used to interpret the coefficient values—(1) ICC < 0.20: slight agreement; (2) 0.21–0.40: fair agreement; (3) 0.41–0.60: moderate agreement; (4) 0.61–0.80: substantial agreement; (5) >0.80: almost perfect agreement (Montgomery et al. 2002).

Very rarely the variables rate of change reached 2 m/s and 2 m/s^2^ for speed and acceleration, respectively. Thus, a sampling rate of 2 Hz was considered an appropriate frequency to capture the time-scale of variability of all the quantities measured.

## Results

**Fig. 1:**
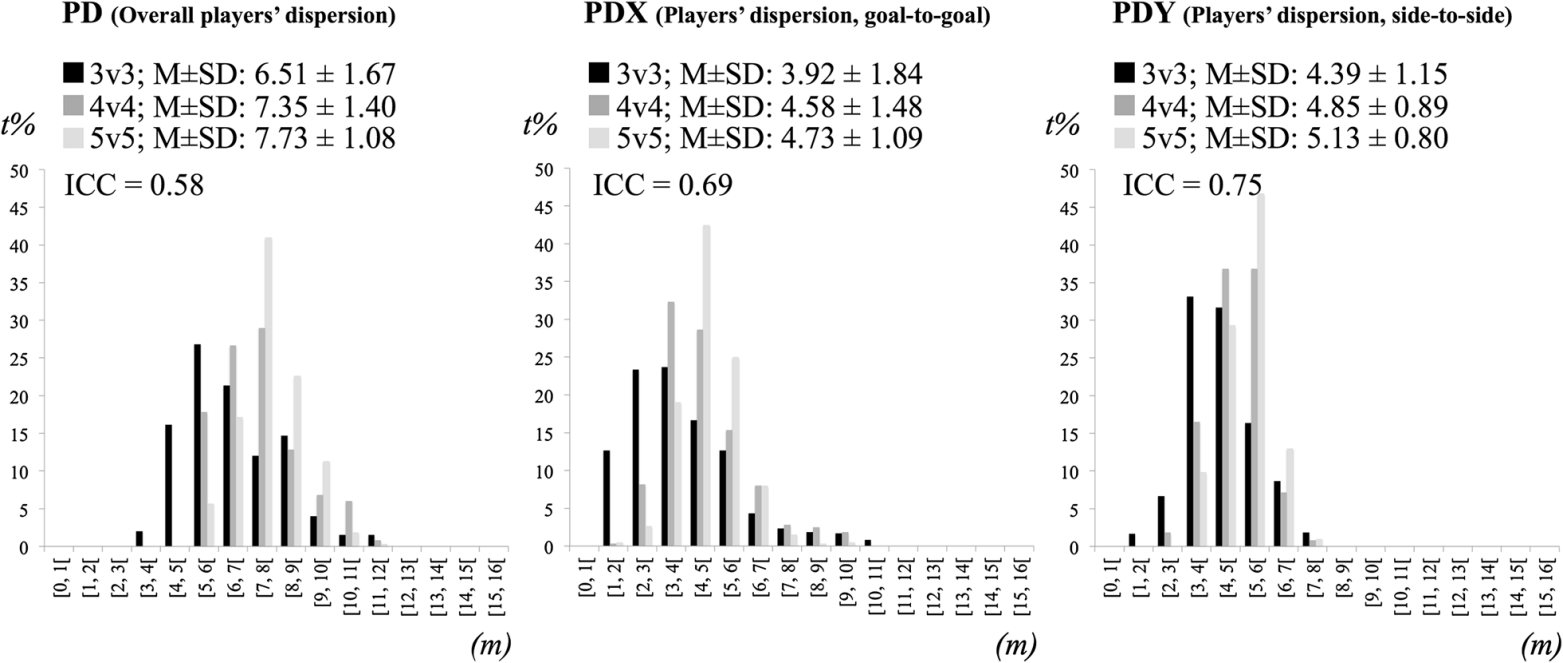
Means ± SD and percentage of time frequencies on values of overall players’ dispersion (PD), players’ dispersion in the goal-to-goal direction (PDX) and players’ dispersion in the side-to-side direction (PDY) across conditions (*m* meters, *t* *%* percentage of time)

Figure 1 shows the time frequencies (in percentages) of players’ dispersion, players’ dispersion in the goal-to-goal direction and players’ dispersion in the side-to-side direction plus mean ± standard deviation values (M ± SD) of each variable across treatments. It is possible to verify that players’ dispersion increases about 1 *m* across conditions (see mean values also represented in Fig. 1). The magnitude of that increase is similar for both directions (≈1 m of difference from the 3v3 to the 5v5 treatments for players’ dispersion in the goal-to-goal direction and players’ dispersion in the side-to-side direction). However, the values of players’ dispersion in the side-to-side direction were slightly larger than values of players’ dispersion in the goal-to-goal direction for all treatments. Data dispersion of players’ dispersion, players’ dispersion in the goal-to-goal direction and players’ dispersion in the side-to-side direction illustrated in the histograms of Fig. 1 followed these results. For instance, it is possible to verify that in the 5v5 treatment, more than 40 % of the match duration was played inside the 7–8 m interval for players’ dispersion, 4–5 m interval for players’ dispersion in the goal-to-goal direction and 5–6 m interval for players’ dispersion in the side-to-side direction. The consistency analysis yielded low to moderate ICC values for players’ dispersion (ICC = 0.58, CI 95 % = 0.16–0.87), players’ dispersion in the goal-to-goal direction (ICC = 0.69, CI 95 % = 0.34–0.9) and players’ dispersion in the side-to-side direction (ICC = 0.75, CI 95 % = 0.35–0.94).

**Fig. 2:**
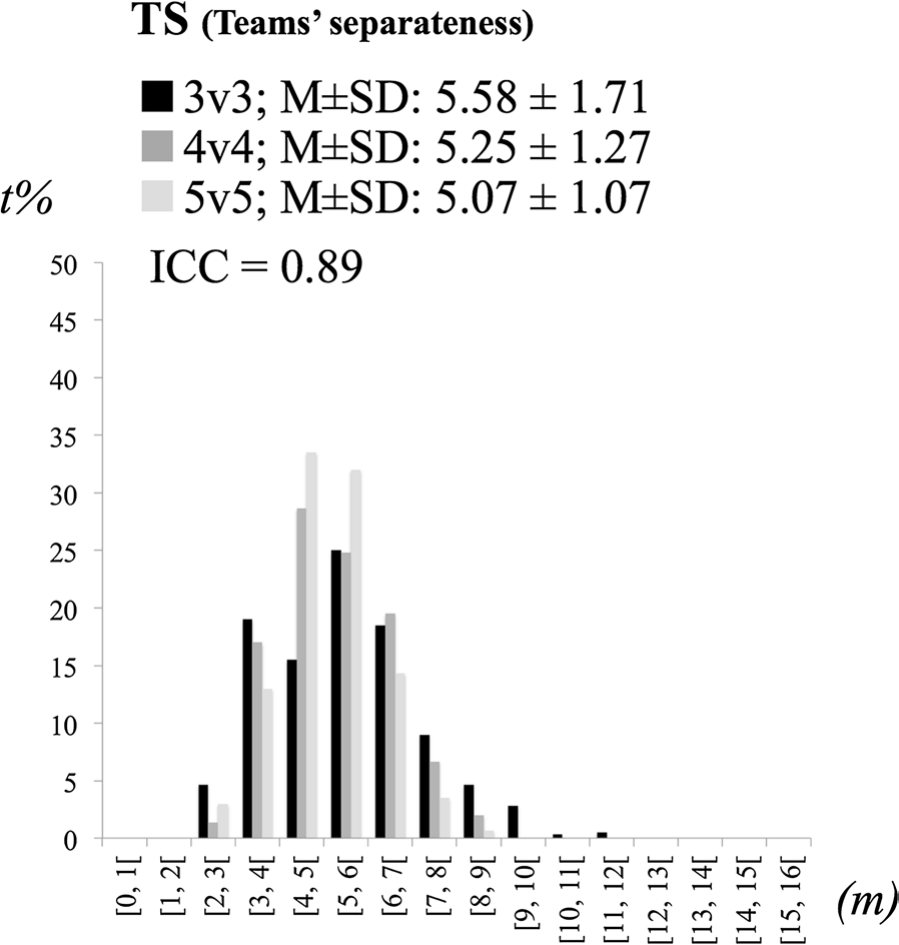
Means ± SD and percentage of time frequencies on values teams’ separateness (TS) across conditions (*m* meters, *t* *%* percentage of time)

The values of teams’ separateness were maintained relatively identically across treatments (Fig. 2). The ICC result obtained confirms the high levels of consistency of this variable across treatments (ICC = 0.89, CI 95 % = 0.71–0.96).

**Fig. 3:**
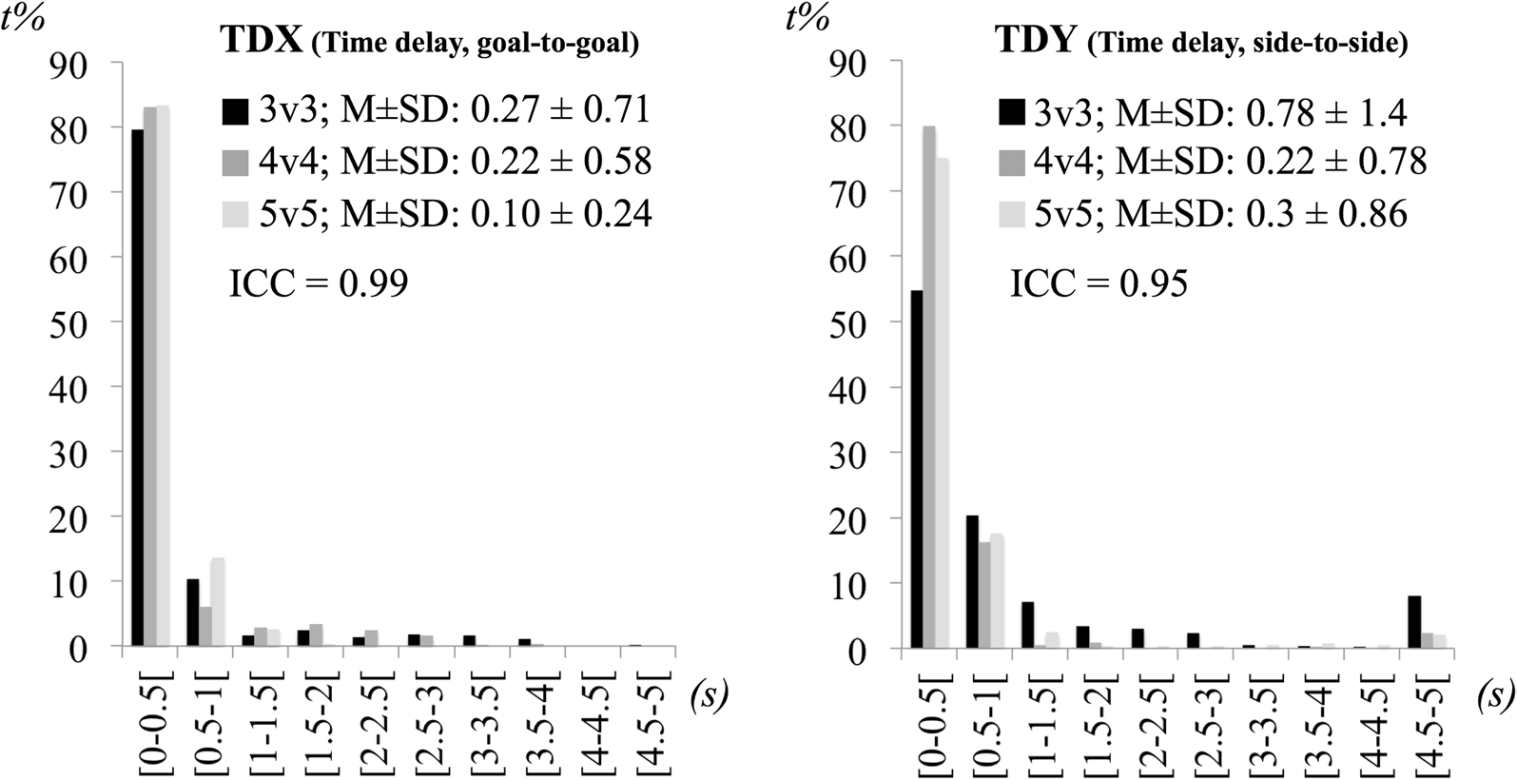
Means ± SD and percentage of time frequencies on values of teams’ dispersion on the goal-to-goal (TDX) and side-to-side (TDY) direction across conditions (*s* seconds, *t* *%* percentage of time)

With regard to the team dispersion in the goal-to-goal and side-to-side directions, Fig. 3 (upper panels) shows that teams reacted to each other approximately within 0 to 1 s. But, in most cases, teams reacted instantaneously to each other’s movements. All ICCs were very high (team dispersion in the goal-to-goal direction: ICC = 0.99, CI 98 % = 0.98–0.99; team dispersion in the side-to-side direction: ICC = 0.83, CI 95 % = 0.59–0.95) suggesting an almost perfect agreement for max lags frequencies across SSCGs and indicating strong consistency of team dispersion between SSCG treatments for both the goal-to-goal and side-to-side directions.

**Fig. 4:**
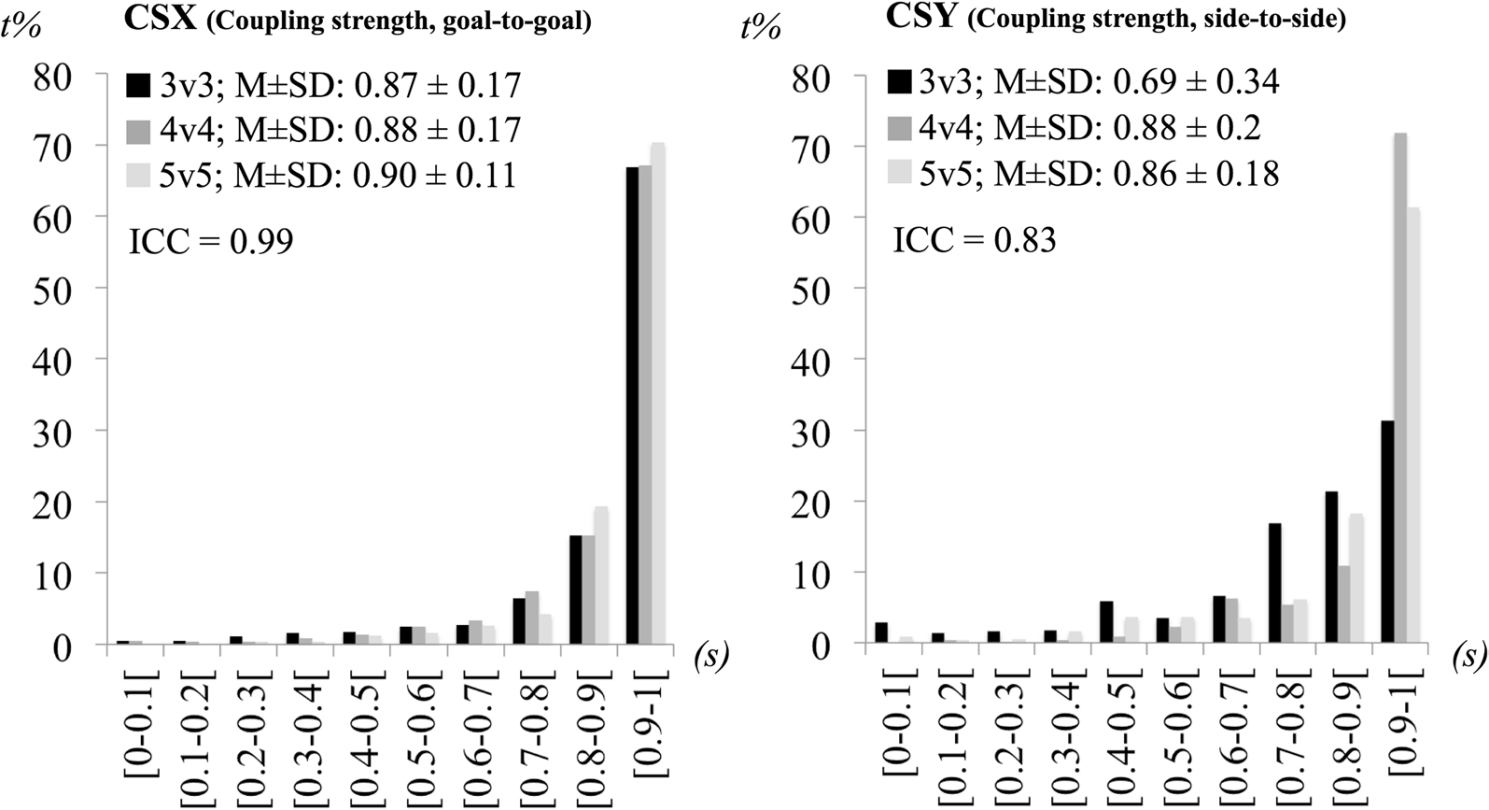
Percentage of time frequencies on values of coupling strength on the goal-to-goal (CSX) and side-to-side directions (CSY) across conditions (*s*—seconds, *t* *%*—percentage of time)

The values of coupling strength were also high (see Fig. 4) and very similar for both directions (goal-to-goal and side-to-side).

**Fig. 5:**
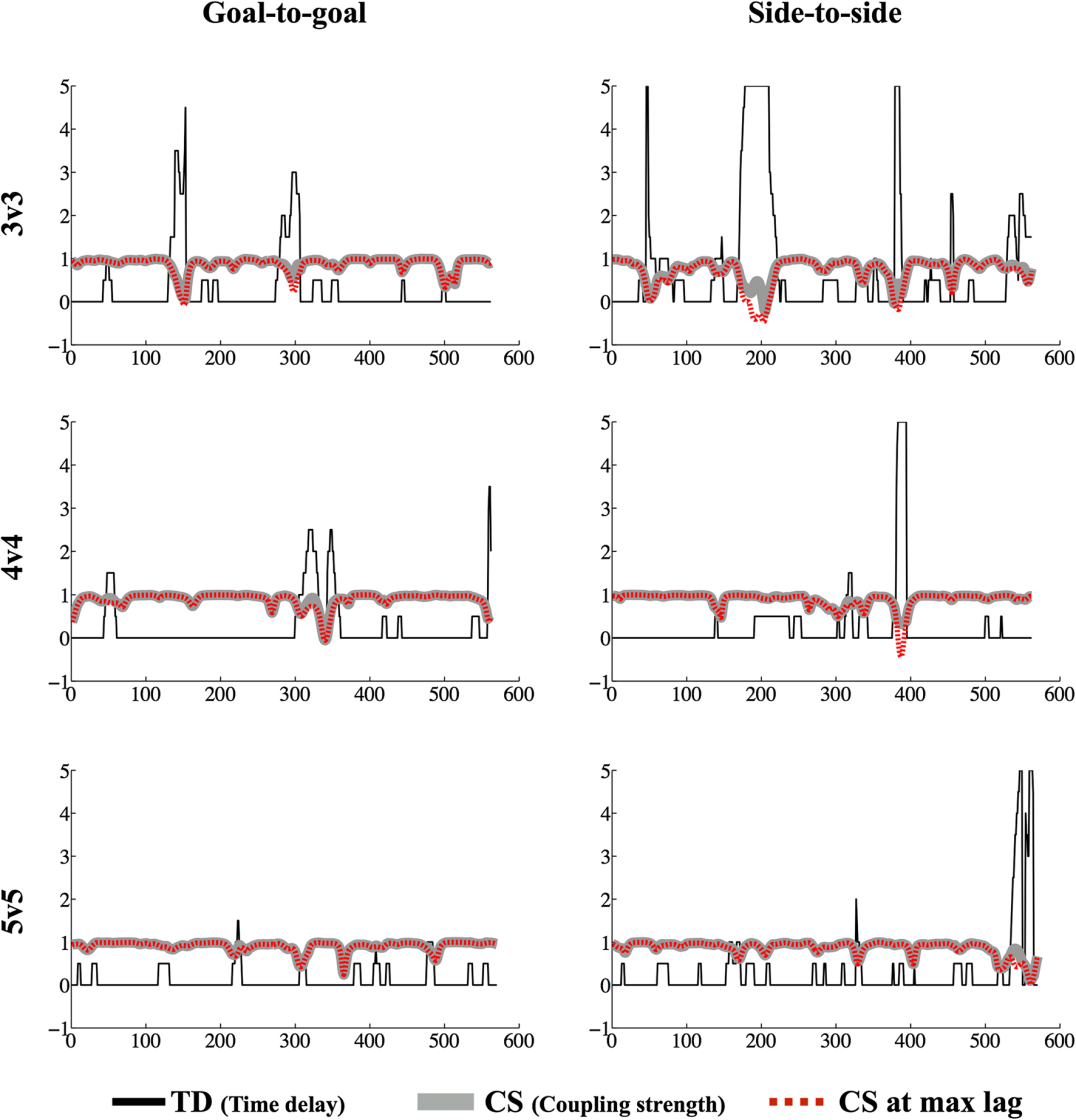
Plots of time delay (TD) and coupling strength (CS) at the max- and zero-lags, according to field direction. The y-axis represents both time (in s) and correlation coefficient values (*r*). The x-axis represents each SSCG time duration

In general, *r* values were above 0.8, reflecting a strong coupling between the teams’ centroids in both field directions. There was an exception, however, on coupling strength in the side-to-side direction in the 3v3 treatment where a value of 0.69 was found. The ICC values were also large (ICC = 0.99, CI 95 % = 0.991–0.999 and ICC = 0.83, CI 95 % = 0.59–0.95 for coupling strength in the goal-to-goal and side-to-side directions, respectively) indicating consistent coupling values across treatments. Figure 5 shows the time evolving measures of coupling strength in each axis at the zero-lag and at the max-lags, as well as the max-lags represented across time. It is apparent that, for most of the time, the strongest couplings occurred at the zero-lag, which was often coincident with the max-lag (the lines at the max-lags and zero lags are often superimposed for both variables). It is also important to note in Fig. 3 that most of the time delays that differed from zero seconds were very short in duration, and that these delays occurred more often in the 5v5 treatment, for team dispersion in both the goal-to-goal and side-to-side directions, and in the 3v3 treatment for the side-to-side direction only.

## Discussion

In this study we adopted a complex systems orientation to analyse effects of different RSP values on processes of inter-team coordination during SSCGs in a group of youth football players (U-15 years). The RSP values were manipulated by increasing the number of players involved in the games while maintaining pitch dimensions constant. The functional spatial–temporal order of inter-team coordination was investigated by comparing the players’ dispersion, the space separating nearest opposing players and the time delay and coupling strength between the teams’ movements.

Results showed that as the RSP decreased, the players of both teams tended to spread on field. This was clear when analysing the overall players’ dispersion and its direction. The players’ dispersion values increased in a similar magnitude for both the goal-to-goal and side-to-side directions. The consistency of these measures across SSCGs seemed to be moderate to low, meaning that adding players to each team impacted on their dispersion in the field. An increase in the players’ spread during SSCGs varying in pitch dimensions was also recorded by Frencken et al. (2013) by measuring the surface areas of both teams.

Regarding teams’ separateness, results showed that the average radius of opposition free for each player did not change across SSCGs. Mean values remained roughly the same across treatments as well as its consistency, despite the reduction of the RSP. In this sense, the results observed for this group of players are in disagreement with the assumption that exists in many coaching manuals (e.g., Lucchesi 2002) that implementing a reduction of the RSP forces them to play with smaller distance values to immediate opponents. The participants forming the collective system in this age range seemed to have the capacity to reorganize themselves in order to maintain a comfortable level of space to their nearest opponent, as the RSP value decreased. In this study, a decrease of 40 % (≈67 m^2^) in the RSP, obtained between the 3v3 and 5v5 conditions, did not promote such space reductions between opposing players. Possibly, this might emerge at larger ratio relations between pitch size and player numbers other than those used in this study. This finding emphasizes the importance, for coaches, of clearly evaluating what tactical adaptations may emerge when they manipulate RSP through player number manipulations.

Curiously, in the work of Frencken et al. (2013) significantly different inter-team distance values were observed according to different RSPs. However, in their study the RSP was manipulated by varying pitch sizes while keeping constant the number of players. In that case it seems that the unaltered collective system analysed (4v4 plus goalkeepers) co-adapted to the changing task constraints (pitch dimensions) by displaying different intra- and inter-team emergent behaviours. In the present study, adding extra players to a team (i.e., altering the properties of the collective system) playing in a constant playing area seemed to have had implications for the reorganization of system components (i.e., the players) to maintain similar interacting inter-team behavioural patterns. Increases in players’ dispersion, players’ dispersion in the goal-to-goal direction and players’ dispersion in the side-to-side direction, reported previously, are probably associated with the non-changing teams’ separateness. As the number of players increased, the players spread out in order to maintain the same distances to nearest opponents. We believe this result to depict the degenerate behaviour of sports teams as complex adaptive systems (Davids and Araújo 2010; Edelman and Gally 2001). Even though the informational constraints imposed by different SSCGs structures changed, teams have co-adapted to maintain similar performance behaviours across SSCGs in order to satisfy functional task solutions. In this study, similar distances to opponent players were preserved, independently of the number of players performing in the SSCGs.

The different RSP values did not impact on the teams’ movements in coupling strength in the goal-to-goal and side-to-side directions, which, generally, were highly synchronous for most of the match time in all SSCG treatments, independent of playing direction. Similar results have been reported in studies of professional matches, where teams have been observed to move synchronously and in a tightly coupled manner (Lames et al. 2010; Yue et al. 2008; Silva et al. 2015) during performance, as well as in SSCGs (Frencken et al. 2011).

With regard to the response delays between the teams’ movements, in most situations it was practically non-existent (or no longer than one second) and unaffected by the different RSP values. The centroids were synchronized without practically any time delay for most of the SSCGs time duration. Nevertheless, these results must be interpreted with caution, as the impact provided by a 0.5 or 1-s delay on match performance was not evaluated in this study. The small number of times that centroids got more than a 0.5-s advantage could be sufficient to destabilize the teams’ symmetry in specific game contexts, leading to the emergence of critical states where the balance between the opposing teams could be perturbed. In these situations the system can transit to other performance outcomes, like the creation of goal scoring opportunities, and thus, impact significantly on the outcome of the match. Such behaviours have been previously observed in several team ball sports (Passos et al. 2008a, 2009; Davids et al. 2006; Araújo et al. 2004).

It is also important to emphasize that most of the reported time-delays were short in duration and were associated with decreases in the teams’ coupling strength. The result of such interpersonal coordination modes on symmetry breaking between teams and its impact on the match outcomes are unknown and needs to be addressed in future research (e.g., questions of importance: are shooting opportunities or transitions in ball possession associated with time delays in teams’ synchronization?). The 5v5 condition seemed to be more prone to such events, especially in teams’ synchrony from side to side.

Despite few observable differences at the teams’ separateness, team dispersion in the goal-to-goal direction, team dispersion in the side-to-side direction, coupling strength in the goal-to-goal direction and coupling strength in the side-to- side direction levels, the existence of an extra number of players in larger SSCG formats may promote more passing options which can impact on the players’ decisions and lead to the emergence of different tactical patterns that could not be captured by the variables used in this study. The analysis of the underlying complexity of the ball movement characteristics and the players’ action zones constitute promising possibilities for future studies to capture such behaviours. Another possible limitation of this study is the order in that the SSCGs were played (i.e., first the 3v3, then the 4v4 and finally the 5v5). More studies are needed using a randomized SSCGs order to its effects on performance.

## Conclusions

In sum, in this study, we observed emergent tactical behaviours of teams as they adapted to decreases in RSP values through increases in players’ dispersion in order to maintain space (i.e. constant comfortable distances to nearest opponents). This did not change the coupling strength of movements between teams in any field direction, nor the existing time delay between them. This change in inter-individual coordination (i.e., players’ dispersion) to satisfy similar inter-team behaviours (i.e., teams’ separateness, coupling strength and time delay) across treatments was associated with the collective adaptive system’s degeneracy.

Following these results, it is suggested that the manipulation of player numbers as a pedagogical strategy to alter inter-team distances and spatial–temporal relations for specific tactical purposes (e.g., increasing pressure on attacking players by reducing space and time for decision-making) should be carefully analysed. Such changes might occur at different ratio relations of pitch size/player numbers than those used by practitioners. It is crucial for practitioners to understand at which point the manipulation of the ratio pitch size/player numbers can be used to stimulate specific tactical behaviours. The manipulation of informational constraints to shape tactical behaviour may be an asset only if the practitioner controls minimally the behaviours that are intended to emerge during the SSCGs. This justifies the need for more studies on SSCGs focusing tactical behaviour variations according to manipulation of player numbers.

The use of different age groups and expertise levels may impact on the results of this experiment. Therefore, it is suggested that future studies could replicate this experiment (or similar) with a higher number of participants of diversified age groups, skills and genders. It would also be interesting to analyse other numerical relations using the actual pitch dimension, like for example, 6v6, 7v7, to try and find an RSP borderline from which teams, as collective systems, would display different behavioural trends.

## References

[CR1] Araújo D, Davids K, Bennett S, Button C, Chapman G, Williams M, Hodges N (2004). Emergence of sport skills under constraints. Skill acquisition in sport research, theory and practice.

[CR2] Balague N, Torrents C, Hristovski R, Davids K, Araújo D (2013). Overview of complex systems in sport. J Syst Sci Complex.

[CR3] Boker SM, Xu M, Rotondo JL, King K (2002). Windowed cross-correlation and peak picking for the analysis of variability in the association between behavioral time series. Psychol Methods.

[CR4] Chow JY, Davids K, Button C, Shuttleworth R, Renshaw I, Araújo D (2006). Nonlinear pedagogy: a constraints-led framework for understanding emergence of game play and movement skills. Nonlinear Dynamics Psychol Life Sci.

[CR5] Coutts AJ, Duffield R (2010). Validity and reliability of GPS devices for measuring movement demands of team sports. J Sci Med Sport.

[CR6] Davids K, Araújo D (2010). The concept of “Organismic Asymmetry” in sport science. J Sci Med Sport.

[CR7] Davids K, Glazier P (2010). Deconstructing neurobiological coordination: the role of the biomechanics-motor control nexus. Exerc Sport Sci Rev.

[CR8] Davids K, Glazier P, Araújo D, Bartlett R (2003). Movement systems as dynamical systems: the functional role of variability and its implications for sports medicine. Sports Med.

[CR9] Davids K, Araújo D, Shuttleworth R, Reilly T, Cabri J, Araújo D (2005). Applications of dynamical system theory to football. Science and football V.

[CR10] Davids K, Button C, Araújo D, Renshaw I, Hristovski R (2006). Movement models from sports provide representative task constraints for studying adaptive behaviour in human movement studies. Adapt Behav.

[CR11] Davids K, Button C, Bennett S (2008). Dynamics of skill acquisition: a constraints-led approach.

[CR12] Davids K, Araújo D, Correia V, Vilar L (2013). How small-sided and conditioned games enhance acquisition of movement and decision-making skills. Exerc Sport Sci Rev.

[CR13] Dellal A, Drust B, Lago-Penas C (2012). Variation of activity demands in small-sided soccer games. Int J Sports Med.

[CR14] Duarte R, Araújo D, Gazimba V, Fernandes O, Folgado H, Marmeleira J, Davids K (2010). The ecological dynamics of 1v1 sub-phases in association football. Open Sports Sci J.

[CR15] Duarte R, Araújo D, Correia V, Davids K (2012). Sport teams as superorganisms: implications of biological models for research and practice in team sports performance analysis. Sports Med.

[CR16] Duarte R, Araújo D, Folgado H, Esteves P, Marques P, Davids K (2013). Capturing complex, non-linear team behaviours during competitive football performance. J Syst Sci Complex.

[CR17] Edelman G, Gally J (2001). Degeneracy and complexity in biological systems. Proc Natl Acad Sci USA.

[CR18] Folgado H, Lemmink KAPM, Frencken W, Sampaio J (2012). Length, width and centroid distance as measures of teams tactical performance in youth football. Eur J Sport Sci.

[CR19] Fradua L, Zubillaga A, Caro Ó, Iván Fernández-García Á, Ruiz-Ruiz C, Tenga A (2013). Designing small-sided games for training tactical aspects in soccer: extrapolating pitch sizes from full-size professional matches. J Sports Sci.

[CR20] Frencken W, Lemmink K, Delleman N, Visscher C (2011). Oscillations of centroid position and surface area of soccer teams in small-sided games. Eur J Sport Sci.

[CR21] Frencken W, van der Plaats J, Visscher C, Lemmink K (2013). Size matters: pitch dimensions constrain interactive team behaviour in soccer. J Syst Sci Complex.

[CR22] Headrick J, Davids K, Renshaw I, Araújo D, Passos P, Fernandes O (2012). Proximity-to-goal as a constraint on patterns of behaviour in attacker-defender dyads in team games. J Sports Sci.

[CR23] Johnston R, Watsford M, Pine M, Spurrs R, Sporri D (2013). Assessment of 5 Hz and 10 Hz GPS units for measuring athlete movement demands. Int J Perform Anal Sport.

[CR24] Lames M, Ertmer J, Walter F (2010). Oscillations in football—order and disorder in spatial interactions between the two teams. Int J Sport Psychol.

[CR25] Lucchesi M (2002). Pressing.

[CR26] Montgomery A, Graham A, Evans P, Fahey T (2002). Inter-rater agreement in the scoring of abstracts submitted to a primary care research conference. BMC Health Serv Res.

[CR27] Passos P, Araújo D, Davids K, Gouveia L, Milho J, Serpa S (2008). Information-governing dynamics of attacker-defender interactions in youth Rugby Union. J Sports Sci.

[CR28] Passos P, Araújo D, Davids K, Shuttleworth R (2008). Manipulating constraints to train decision making in Rugby Union. Int J Sports Sci Coach.

[CR29] Passos P, Araújo D, Davids K, Gouveia L, Serpa S, Milho J (2009). Interpersonal pattern dynamics and adaptative behavior in multiagent neurobiological systems: conceptual model and data. J Mot Behav.

[CR30] Platt D, Maxwell A, Horn R, Williams M, Reilly T (2001). Physiological and technical anlysis of 3v3 and 5v5 youth football matches. Insight.

[CR31] Seifert L, Button C, Davids K (2013). Key properties of expert movement systems in sport. Sports Med.

[CR32] Serra-Olivares J, González-Víllora S, García-López LM (2015). Effects of the modification of task constraints in 3 vs. 3 small-sided soccer games. S Afr J Res Sport Phys Educ Recreat.

[CR33] Serra-Olivares J, González-Víllora S, García-López LM, Araújo D (2015). Game-centred approaches’ pedagogical principles: exploring task constraints in youth soccer. J Hum Kinet.

[CR34] Silva P, Garganta J, Araújo D, Davids K, Aguiar P (2013). Shared knowledge or shared affordances? Insights from an ecological dynamics approach to team coordination in sports. Sports Med.

[CR35] Silva P, Travassos B, Vilar L, Aguiar P, Davids K, Araújo D, Garganta J (2014). Numerical relations and skill level constrain co-adaptive behaviors of agents in sports teams. PLoS ONE.

[CR36] Silva P, Chung D, Carvalho T, Cardoso T, Davids K, Araujo D, Garganta J (2015). Practice effects on intra-team synergies in football teams. Hum Mov Sci.

[CR37] Sinnott RW (1984). Virtues of the Haversine. Sky Telescope.

[CR38] Travassos B, Araújo D, Duarte R, McGarry T (2012). Spatiotemporal coordination behaviors in futsal (indoor football) are guided by informational game constraints. Hum Mov Sci.

[CR39] Travassos B, Gonçalves B, Marcelino R, Monteiro R, Sampaio J (2014). How perceiving additional targets modifies teams’ tactical behavior during football small-sided games. Hum Mov Sci.

[CR40] Vilar L, Araújo D, Davids K, Travassos B (2012). Constraints on competitive performance of attacker-defender dyads in team sports. J Sports Sci.

[CR41] Vilar L, Duarte R, Silva P, Chow JY, Davids K (2014) The influence of pitch dimensions on performance during small-sided and conditioned soccer games. J Sports Sci. doi:10.1080/02640414.2014.918640 10.1080/02640414.2014.918640 24915106

[CR42] Withagen R, Poel HJd, Araújo D, Pepping G-J (2012). Affordances can invite behaviour: reconsidering the relationship between affordances and agency. New Ideas Psychol.

[CR43] Yue Z, Broich H, Seifriz F, Mester J (2008). Mathematical analysis of a soccer game. Part II: energy, spectral and correlation analyses. Stud Appl Math.

